# Microbial Diagnostic Microarrays for the Detection and Typing of Food- and Water-Borne (Bacterial) Pathogens

**DOI:** 10.3390/microarrays1010003

**Published:** 2011-10-14

**Authors:** Tanja Kostić, Angela Sessitsch

**Affiliations:** 1 AIT Austrian Institute of Technology GmbH, Health & Environment Department, Bioresources Unit, Konrad Lorenz Strasse 24, A-3430 Tulln an der Donau, Austria; 2 Christian Doppler Laboratory for Molecular Food Analytics, University of Veterinary Medicine, Veterinärplatz 1, A-1210 Vienna, Austria

**Keywords:** microbial diagnostic microarray, foodborne pathogens, waterborne pathogens, pathogen detection

## Abstract

Reliable and sensitive pathogen detection in clinical and environmental (including food and water) samples is of greatest importance for public health. Standard microbiological methods have several limitations and improved alternatives are needed. Most important requirements for reliable analysis include: (i) specificity; (ii) sensitivity; (iii) multiplexing potential; (iv) robustness; (v) speed; (vi) automation potential; and (vii) low cost. Microarray technology can, through its very nature, fulfill many of these requirements directly and the remaining challenges have been tackled. In this review, we attempt to compare performance characteristics of the microbial diagnostic microarrays developed for the detection and typing of food and water pathogens, and discuss limitations, points still to be addressed and issues specific for the analysis of food, water and environmental samples.

## 1. Introduction

Microbiological analysis of food, water and environmental samples is an important aspect of public health involving a great number of samples to be analyzed daily. These analyses are, to a great extent, performed by responsible state laboratories, by private laboratories or by companies as a quality control measure. Methods used mostly rely on cultivation of target pathogens or indicator microorganisms on certain media and are thus simple, but require days or, in some cases, weeks until results are obtained. In the last decades molecular methods have been developed, most importantly PCR-based techniques, which rapidly detect DNA of a specific microorganism. PCR is a very convenient technique for the detection of single microbes and multiplex-PCR systems may be applied for the detection of few target organisms. Nevertheless, the multiplexing potential is limited and thus the approach is sub-optimal when parallel detection of all relevant pathogens is needed. This high degree of parallelism can be achieved by the use of microarrays. DNA-based microarrays may contain hundreds to thousands of different, selective probe molecules, which are designed to hybridize with their respective target DNA; *i.e.*, the DNA of those microorganisms to be detected in a single assay. Gene expression analysis was the first issue to be addressed by microarray technology [1], however, recent years have witnessed widespread expansion to other application fields such as comparative genomics, sequence analysis and diagnostics. This development has been supported and accompanied by further technological achievements, e.g., by different kinds of probe molecules, surface chemistries, amplification/labeling methods and microarray platforms. These developments have been reviewed extensively [2,3,4,5,6,7] and therefore only the most critical parameters related to the development of microbial diagnostic microarrays (MDMs) will be discussed in this review. 

Specific criteria apply to the analysis of food and water samples. One key aspect to consider is that the target microorganisms have to be detected in a background microflora, whose composition and abundance will vary greatly and depend on the type of food/water sample. Furthermore, food matrices vary and will substantially influence the analytical process. It should also be considered that routine laboratories have to handle a large number of samples and that analysis costs play a more important role than, e.g., for clinical samples. This review will focus on the MDMs that have been developed up to date for food and water analysis (for an overview see also Table 1) and discuss their potential to fulfill the analytical requirements.

## 2. Microbial Diagnostic Microarrays (MDMs)

### 2.1. Selection of Diagnostic Markers

Molecular diagnostic markers are DNA fragments which allow identification and/or characterization of organisms. Issues that have to be considered when selecting diagnostic markers are: (i) distribution of the marker in the target microorganisms; (ii) differentiation potential; (iii) informational content (phylogenetic affiliation *vs.* pathogenicity); and (iv) sequence availability. Conventional phylogenetic markers (e.g., ribosomal RNA genes, *gyrB* or *rpoB*) are ubiquitously distributed among all bacterial species. In general, it can be said that the degree of conservation is disproportional to the differentiation potential, *i.e.*, highly conserved genes such as the 16S rRNA gene allow the design of genus- and in some cases species-specific probes, whereas less conserved markers such as the *gyrB* gene allow, in most cases, the design of species-specific probes. However, the 16S rRNA gene is the most frequently analyzed bacterial marker and therefore the most extensive sequence database is available enabling in-depth phylogenetic analysis of bacteria. The availability of a sequence database as well as its quality and size strongly influence probe design as highly specific probes can only be based on an appropriate set of sequences. Another important issue is the informational content of the selected diagnostic markers. Especially when considering the analysis of food, water and environmental samples, it is important to discriminate between the presence of ubiquitous bacterial species and their potentially pathogenic relatives (e.g., *E. colivs.* EHEC). For this purpose, e.g., virulence genes may be a good choice. Alternatively, increasing availability of whole genome sequences allows for the *in-silico* determination of novel diagnostic markers [8]. 

### 2.2. Probe Selection

MDMs utilize in most cases three kinds of probes: short and long oligonucleotides and PCR amplicons. Short oligonucleotides have high differentiation potential (1–2 mismatches) and are therefore indicated in connection with highly conserved phylogenetic markers. As a disadvantage they display lower binding capacity and thus necessitate utilization of PCR amplification. Long oligonucleotide probes and PCR products on the other hand are characterized by a lower discrimination potential (80–85% sequence homology will suffice for a positive hybridization event) but higher binding capacity and can thus also be combined with more generic amplification approaches (e.g., whole genome amplification, WGA). In addition to the probe length, mismatch position also plays a major role in hybridization specificity. These effects were nicely described by Letowski and co-workers [9]. 

### 2.3. Selection of DNA Amplification/Labeling Methods

Amplification of targeted nucleic acids has a significant effect on the sensitivity of the diagnostic system. PCR amplification is the most sensitive method; however, it has limited multiplexing potential and is therefore not very compatible with systems that employ numerous targets (e.g., virulence gene arrays). As alternatives, whole genome or linear amplifications may be considered. A comprehensive study on the effects of selected DNA amplification was published by Vora and co-workers [10]. 

Another parameter that should be considered is the utilization of fluorescence *vs.* colorimetric labeling approaches. Especially in the case, where costs are an important issue colorimetric labeling can be of advantage. A comparison of sample-labeling techniques was performed and published by Baggerly and co-workers [11]. 

### 2.4. Selection of the Assay Format

A planar glass slide with different surface modifications that allow covalent binding of probe molecules is the conventional microarray format widely accepted in research labs. However, this format necessitates skilled handling and does not meet high acceptance in routine laboratories. Therefore, alternative formats have been developed. Microarrays in tubes or 96-well plates for example represent user-friendly formats, often used in commercialized products for the routine market (e.g., StaphyType96 from Alere Technologies GmbH (previously ClonDiag; [12]), Legionellachip® from LEGYON® [13], CLART® FluAVir or CLART® FluAVir from Genomica [14]. Bead-coupled microarrays represent another alternative (e.g., xMAP technology from Luminex [15,16])). 

Even though, all these elements are essential building blocks of a good MDM, most challenging and most important task is to enable and demonstrate that the developed tool can perform as required in the scope of a complete analytical chain. This review will attempt to answer the question if currently available MDMs for pathogen detection in food, environmental and water samples are really ready for this challenge and if some bottlenecks can still be identified. 

## 3. Application of Microbial Diagnostic Microarrays for Pathogen Detection in Food, Environmental and Water Samples

### 3.1. 16S rRNA Gene-Based MDMs

The 16S rRNA gene serves as an evolutionary clock and taxonomic marker in bacterial systematics [17,18]. Consequently, the 16S rRNA gene is a commonly used marker for MDMs. The ubiquitous distribution, the presence of both highly conserved and variable regions, and the availability of an extensive and publicly accessible database are appealing reasons to choose 16S rRNA genes as a diagnostic marker. Furthermore, multiple copies of 16S rRNA genes are present in the majority of bacterial genomes [19], which contributes to enhanced sensitivity. However, due to the high conservation degree, the resolution potential of the 16S rRNA gene is limited and can therefore prevent the differentiation of closely related species. Few representative 16S rRNA gene-based MDMs for pathogen detection in food, environmental and water samples will be discussed in more detail.

Wang and co-workers [20] reported on the development and application of a 16S rRNA gene-based microarray for the detection of food-borne pathogens. Twenty-eight short oligonucleotide probes were designed (including positive and negative control probes) based on 128 bacterial 16S rDNA sequences (ranging from 1 to >10 sequences per targeted species). The authors applied a hierarchical probe design including universal bacterial probes as well as genus- and species-specific probes (single probes for each target). The specificity of the microarray was validated using more than 200 strains of target organisms (inclusivity), exclusivity tests have not been performed. Since microarray could not differentiate between strains belonging to *Shigella* spp./*E. coli* and *Salmonella* spp. a second microarray, based on species specific *virA* and *invA* genes, was developed. The array was further validated by using 115 bacteria isolated from food samples, in 112 cases (97.4%) microarray results agreed with those obtained by employing conventional methods. The absolute limit of detection was determined with spiked food samples and was reported to be at the level of 10^1^ to 10^3^ cfu/g food. There is no information on the relative sensitivity of this system. 

A 16S rRNA gene-based MDM for the detection of wastewater bacterial pathogens was developed by Lee and co-workers [21]. This microarray consists of 62 short oligonucleotide probes targeting 38 bacterial species, 4 genera, and 1 family, all known to include pathogens. Probe coverage ranged from one (e.g., *Vibrio cholerae*) to five per target (e.g., *Helicobacter pylori*). Probe specificity was confirmed only by *in-silico* analysis (BLAST search of NCBI database) and the authors did not report on any *in vitro* validation. The sensitivity of the microarray was determined using dual genomic DNA dilutions of *Aeromonas hydrophila* and *E. coli* and was found to be 1% (relative sensitivity) or 10^4^ copies of rRNA genes (absolute sensitivity, corresponding to 10^3^ cells). To demonstrate the applicability of the proposed detection system the authors used wastewater samples collected from two municipal wastewater treatment plants (both prior and after the disinfection step). Microarray results were compared to those obtained by species-specific TaqMan qPCR and in general good agreement between both methods was observed. Minor observed differences were attributed to the different number of detection probes and/or target genes. 

A major drawback of 16S rRNA gene-based arrays is the limited differentiation potential of the 16S rRNA marker gene. This can be especially problematic if detection is performed on complex matrices such as wastewater or food. For example, a more detailed investigation (using NCBI BLAST tool; [22]) of published *V. cholerae* 16S rRNA gene-based probes revealed a rather questionable specificity. Probe Vchol16S 5’ AAGTTAATACCTTAATCATTTGACG 3’ [21] showed 100% homology with *V. mimicus* and *V. albensis* 16S rRNA genes. Probe #19 5’ gctccaccttcgcggtattcgctgccct 3’ [20] was homologous with only one *V. cholerae* 16S sequence in NCBI database (accession no: X76337). Modified probe sequence 5’ gctccacctcgcggtatcgctgccct 3’ that would detect most *V. cholerae* isolates (according to the at this time available sequence data) showed also 100% homology with 16S rRNA genes of a range of other species including *V. mimicus*, *Desulfovibrio vulgaris* and *Colwellia* spp. Similar observations can be made for a range of other published putatively species-specific 16S rRNA probes. These findings indicate the importance of extensive *in vitro* validation that should always include both inclusivity (multiple strains of each targeted species) and exclusivity tests (strains of closely related species).

Cremonesi and co-workers [23] developed a 16S rRNA gene-based microarray employing a ligation detection reaction (LDR). This approach utilizes the discriminatory power of ligase that joins ends of two discriminatory probes, thus allowing single nucleotide differentiation. LDR probe pairs targeting 15 groups of bacterial pathogens frequently contaminating dairy products were developed and validated using a set of targeted reference strains (including one negative control strain). Validation results confirmed the high differentiation potential of the LDR approach; even though the *E. coli* probe targeted some *Shigella* spp. strains (and was thus kept as a “*E. coli*_et_rel” probe), clear differentiation was demonstrated for different *Streptococcus* species. The performance of the array was additionally evaluated with 50 milk samples. Results obtained with the microarray correlated well with conventional, microbiological analysis, although the array detected contamination with multiple pathogens, which was not the case with standard methods. However, one disadvantage of the LDR system is the high cost of the probes.

The 16S–23S rDNA gene internal transcribed spacer (ITS) was employed as a marker for the development of a MDM for the detection of 10 different pathogenic bacteria associated with powder infant formula (PIF) contamination [24]. One to four probes for every target were designed. In addition to the ITS marker, the *wzy* gene, encoding O antigen polymerase, was used for the detection of *E. coli* O157. Microarray specificity was tested *in vitro* through the analysis of 185 strains (134 target pathogen strains and 51 closely related bacteria). Obtained results allowed the selection of 27 species-specific probes, from the 79 probes initially screened, that enabled correct identification of all test strains. Microarray sensitivity was shown to be target-specific and ranged between 0.001 and 0.1 ng gDNA and 10^4^ cfu/mL in a pure culture. Even though the authors reported reliable detection from binary and ternary mixtures, data on the relative sensitivity of the microarray have not been provided. Analysis of mock spiked PIF samples revealed sensitivity in the range of 1–10 cfu/25 g sample after biological pre-enrichment. A proof-of-principle study was performed with 21 batches of PIF samples obtained from different sources; two samples were positive for one of the tested pathogens whilst the others displayed signals for the positive control only indicating the presence of other non-targeted microorganisms. These results were in 100% agreement with reference methods. This study revealed that the ITS offers a higher resolution potential than the 16S rRNA gene. As the 16S rRNA gene, the ITS is present in multiple copies in most bacterial species. However, far less ITS sequences are available indicating the need for a more thorough *in vitro* validation. 

### 3.2. MDMs Based on Alternative Marker Genes

Advantages and disadvantages of 16S rRNA gene-based bacterial identification have been comprehensively discussed and alternative markers have been suggested [18]. These include universally conserved genes (e.g., *gyrB* or *rpoB*), group-specific functional genes (e.g., *pmoA* or *nirK*), virulence (e.g., set—*S. aureus* eneterotoxin genes) or other target-specific genes such as *iap* (*Listeria* spp. invasion-associated protein). Alternative markers have been often employed for the development of microbial diagnostic microarrays and some representative examples will be discussed.

Maynard and co-workers [25] compared the suitability of 16S rRNA, *cpn60* and *wecE* genes for the detection of waterborne pathogens. The *cpn60* gene codes for GroEL, a highly conserved chaperonin protein. The *wecE* gene is part of the *Enterobacteriaceae*-specific *wec* gene cluster involved in antigen biosynthesis. Utilization of different marker genes resulted in a more complicated analytical process—three different PCR amplifications were required, and differences in respective annealing temperatures did not allow for implementation of multiplex PCR. The specificity of the system was tested using single strains of the microorganisms to be detected (*E. coli*, *S.* Typhimurium and *Y. enterocolitica*). Even though initial results seemed to confirm the predicted probe specificity, a more detailed *in-silico* analysis revealed inherent differentiation problems of 16S rRNA gene-based probes. This was especially the case for putative *Salmonella* spp. serovar-specific probes that were in fact only specific for the reference sequence. For example, BLAST analysis of the *S.* Agona specific probe S-Ss-S.entA-0076-a-S-22 5’ GAAGCAGCTTGCACGTAGCTGA 3’ revealed only partial homology with other available *S.* Agona sequences (e.g., CP001138); in addition the same level of partial homology was observed with a range of other serovars (e.g., *S.* Dublin (CP001144) or *S.* Paratyphi C (CP000857)). More thorough tests were performed on the sensitivity of the assay. Relative sensitivity of the assay was explored by using binary and ternary mixtures of test strains and was found to be at the level of 0.1–1% depending on the organism and gene (the *cpn60* gene displayed a 10-fold higher detection limit than 16S rRNA and *wecE* genes). The absolute sensitivity was in the range of 5 to 50 pg gDNA and corresponded to 10^3^ to 10^4^ genome equivalents. An additional test was performed with gDNA derived from artificially spiked wastewater. The results of this experiment nicely demonstrated the challenges encountered with the application of microarrays on real-life samples. For example, DNA isolated from an un-spiked wastewater sample did not hybridize with the *Salmonella* spp. specific 16S rDNA probe (S-G-Salm-0467-a-S-22) but hybridized with both *S.* Typhimurium-specific *wecE* probes (W-Ss-Salm-0588-a-S-22 and W-Ss-Salm-0497-a-S-22). Furthermore, the detection of *Klebsiella* spp. and *Yersinia* spp. (*wecE* probes W-G-Kleb-unk.-a-S-20 and W-G-Yers-unk.-a-S-23 respectively) was compromised when the wastewater sample was spiked with higher amounts of *S.* Typhimurium gDNA. These effects were probably caused by inherent PCR amplification bias. 

In our laboratory we explored the usability of the *gyrB* gene (encoding the subunit B of the bacterial gyrase) as a marker for the detection of most common food and water-borne pathogens and indicator organisms [26]. The advantage of *gyrB* gene is its better resolution on the species level. However, a major problem was the rather low availability of sequences and ensuing effects on the probe design quality. The implementation of the SSELO (sequence—specific end labeling of oligonucleotides) method [27], a labeling approach which targets only those regions actually used in the hybridization reaction, together with the competitive oligonucleotides enabled differentiation of highly homologous sequences. Specificity of the probe set was tested using a set of reference strains including both target and non target species. Detection sensitivity was assessed using pure cultures and artificial mixes and was found to be in a range of 0.1% (relative sensitivity) and 10^4^ cfu (absolute sensitivity). In a proof-of-principle study the microarray was challenged with artificially spiked food samples. Qualitative results were in good agreement with the standard microbiological reference methods (ISO) and detection sensitivity was demonstrated to be in the range 1–10 cfu/25 g food (with biological pre-enrichment). Increasing availability of *gyrB* sequences and extended *in vitro* testing revealed that the specificity of some probes were sub-optimal (unpublished data; e.g., probe Cperf_1443 5’ AAGAGGGGCTGTGCTTAC 3’ had only 94% homology with a set of *C. perfringens gyrB* sequences) and therefore, also in this case, further optimization of the system is indicated. 

Utilization of more specific alternative marker genes (e.g., virulence or toxin genes) allows for additional typing of the detected microorganisms. For example, Call and co-workers [28] reported on the development of a microarray for the detection and typing of *E. coli* O157:H7. Four virulence loci (intimin, Shiga-like toxins I and II, and hemolysin) were targeted by short oligonucleotide probes (with a coverage of one probe per gene). A multiplex PCR system was developed for the parallel amplification of all target genes, thus simplifying the analytical process. The specificity of the microarray was tested and confirmed by using five isolates of *E. coli* O157:H7 representing different genotypes regarding Shiga-like toxins. Additional *E. coli* controls were also included in the test. The sensitivity of the detection system was 10 fg gDNA used as PCR template. In a proof-of-principle study spiked chicken carcass rinsates were used. In this case the analytical process included an additional IMS (immune-magnetic separation) enrichment step. Reliable detection was demonstrated for 168 cfu/mL spike level. Lower spike levels could not be detected with 100% reliability. 

A more comprehensive detection/typing microarray was described by Sergeev and co-workers [29]. The FDA-1 microarray allowed simultaneous detection of several food-borne pathogens and their virulence factors including *Listeria* spp., *Campylobacter* spp., *S. aureus* enterotoxin genes and *C. perfringens* toxin genes. Robustness of the system was ensured by using both redundant genes and redundant probe sets for each gene. However, this necessitated utilization of multiple PCR amplification reactions (8 single PCRs) resulting in a somewhat complicated analytical set-up. Specificity of the system was tested using a set of target strains and mixtures thereof, *i.e.*, only inclusivity tests were performed. Information on the detection sensitivity is not available. Even though the system appeared to function well during the initial validation process, results with real-life samples have not been published, and thus there is no information on its performance with real samples.

Wilson and co-workers [30] reported on the development of a high density microarray for the detection of 18 pathogenic microorganisms, including 11 bacteria, 5 RNA viruses, and 2 eukaryotes. The microarray contained more than 50,000 short oligonucleotide probes targeting 142 unique diagnostic regions (3 to 10 per pathogen). For each designed probe a mismatch control probe was designed and used for the discrimination between cross-hybridization and true signals. High diversity of both target organisms and diagnostic regions resulted in a complex analytical procedure including multiplex PCR amplifications as well as reverse transcription (RT)-PCR for the detection of viruses. Probe specificity was tested using a representative isolate of each target pathogen. The positive fraction (*i.e.*, the number of positive probes divided by the total number of probes for a given target) for all probe sets was >80%, and thus 80% was set as a cut-off value for positive calls. The absolute sensitivity was determined to be 10 fg gDNA for *Bacillus anthracis*. The relative detection sensitivity was determined by spiking *Francisella tularensis* and *Yersinia pestis* gDNA in the background of air gDNA. At the higher spike level (2.5% relative abundance) all probe sets (8 per pathogen) were positive. At the lower spike level (0.025% relative abundance) detection was dependent on the probe set, for example 5/8 and 3/8 probe sets positive for *F. tularensis* and *Y. pestis* respectively. 

Another interesting pathogen detection microarray was described by Miller and co-workers [31]. This microarray targeted 12 microbial pathogens relevant for water and food safety. Multiple virulence and marker genes were used (1 to 5 genes per targeted pathogens, 35 genes in total) and each gene was targeted by multiple probes (8 to 35 probes per gene, average 17). In total, the microarray consisted of 791 target probes. In addition more than 2000 non-target probes (oligonucleotides targeting genes not amplified by PCR) were included on the microarray. For the PCR amplification of 35 target genes 47 primer pairs were selected and combined into 5 multiplex PCR reactions (9 to 10 primer pairs each). With the exception of *Pseudomonas aeruginosa* each targeted pathogen was included in at least two different multiplex PCRs. Comprehensive validation was performed firstly by using pooled PCR products from individual singleplex PCRs and subsequently pooled PCR products from multiplex PCR amplifications. 720 (91%) targeted probes passed specificity test in the first (singleplex PCR) and 673 (85%) in the second (multiplex PCR) test run. The differences were mostly attributed to the different PCR yields, and most probes that were negative in the multiplex approach were also weak in the singleplex approach. Only 3% of non-targeted probes displayed false positive results. Sensitivity of detection was tested using spiked water samples, and, depending on the pathogen and gene, demonstrated to be in the range of 0.1%−0.01%. 

As discussed above, the utilization of different marker genes frequently necessitates the development of complex multiplex PCR systems whose performance is often difficult to optimize. As an alternative a combination of whole genome amplification and long oligonucleotide probes can be employed. Such an approach was, for instance, employed by Kim and co-workers [32]. In this study a MDM targeting 11 major food-borne pathogens was developed. Comparative genomic analysis was used to identify DNA regions specific for a certain pathogen (and which had low homology to other species) and 10 overlapping 70-mer oligonucleotide probes were selected for each region. Specificity was tested with a set of 68 bacterial strains including the target pathogens and other related bacteria. With the exception of cross-hybridization of *Listeria ivanovii* to *L. monocytogenes* specific probes (closely related virulence locus) and *B. anthracis* and *B. thuringiensis* to *B. cereus* specific probes (all closely related and designated as *B. cereus* group) all targeted organisms were identified correctly, and all non-targeted organisms yielded negative results, thus confirming that comparative genomics is a promising approach to select discriminative markers. Further studies will be needed to determine the sensitivity and to demonstrate the applicability of the system. 

A more comprehensive MDM, that allows identification and characterization of pathogens as well as detection of their antimicrobial resistance genes, was developed by Peterson and co-workers [33]. This MDM targeted 113 virulence genes from 43 pathogenic bacteria, 227 antimicrobial resistance genes conferring resistance to 30 antimicrobials, 99 genes that encode resistance against 20 metals and cover 31 horizontally transferable elements. Each gene was targeted by single 70mer oligonucleotide, and between one and several virulence genes were used to detect individual organisms. The specificity of this probe set was tested with only 7 organisms containing only a sub-set of targeted virulence and antimicrobial resistance genes. Resulting hybridization profiles were highly complex and not completely unambiguous. In cases when species were targeted via multiple virulence genes apparently not all signals are expected (e.g., from 8 probes targeting virulence genes of *Enterococcus* spp. three are positive with *E. faecalis* and only one with *E. faecium*). This makes it difficult to validate potential cross-hybridization events (e.g., such as *Fusobacterium necrophorum* hybridization with one out of two Cryptosporidium spp. specific virulence probes, or *S.* Typhimurium and *E. coli* hybridization with a single *Streptomyces aureofaciens* specific probe). The detection limit was tested with spiked manure samples and was found to be in a range of 10^9^ to 10^10^ cfu/g. The addition of gene-specific primers during the labeling step resulted in a two log improvement of sensitivity. Further improvement in detection limit was achieved by implementing biological enrichment step (detection limit 10^3^ cfu/g). However, taking the number of different targets and the diversity of target organisms into consideration both proposed sensitivity enhancement approaches do not appear to be practicable. 

The sensitivity issue of long oligonucleotide microarrays was also addressed by Sou and co-workers [34]. The developed microarray was limited to only four highly relevant food-borne pathogens, *E. coli* O157:H7, *Salmonella enterica*, *Listeria monocytogenes* and *Campylobacter jejuni* and encompassed 14 virulence genes (on average 2 per pathogen). This allowed for the implementation of a multiplex PCR amplification in the analytical chain and resulted in the absolute sensitivity of 0.1 pg gDNA, corresponding to approximately 20 genome equivalents. An additional advantage of PCR-based amplification is enhanced specificity. A proof-of-principle study was performed with 39 fresh meat samples and microarray results were in good correlation with the conventional microbiological analysis. However, in this case, the analysis was additionally coupled with biological pre-enrichment, which significantly influenced both specificity and sensitivity of detection (e.g., reduction of enrichment time from 20 h to 6 h in multipathogen selective enrichment broth (SEL) resulted in a 10-fold decrease in sensitivity, from 4 cfu/25 g food to 40 cfu/25 g food).

A higher density and multiplexing level can be accomplished by using Affymetrix-based systems. Berthet and co-workers [35] described a highly complex system (2.56 million 25-mer probes) targeting 42 viruses and more than 50 bacterial species, as well as 390 antibiotic resistance and 229 pathogenicity and virulence genes. The analytical approach was based on whole-genome amplification and re-sequencing microarrays. Even though the specificity (e.g., differentiation of Smallpox and Monkeypox virus) and sensitivity (single genome copy absolute of >0.01% relative sensitivity for *Staphylococcus aureus*) of the system were notable, high costs and the need for complex data analysis algorithms lessens the application potential of such systems in food-, water- and environmental monitoring.

### 3.3. MDMs for Typing of Microorganisms

Microarrays are increasingly used for the typing of the microorganisms. In this case, starting material is mostly an isolate and therefore sensitivity is not an issue. However, specificity and resolution have to be very high and these arrays have often to be phenotype-based (e.g., serotyping) and are therefore challenging to decode at the genetic level. Most developments target clinically and epidemiologically important species. 

Anjum and co-workers [36] developed an *E. coli* pathotyping microarray which was established on the ArrayTube platform and included 39 virulence, 7 bacteriocin and 15 control short oligonucleotide probes (coverage: mostly one and up to four probes per gene). Utilization of a linear amplification reaction allowed for a high degree of multiplexing. The specificity of the probe-set was tested with a panel of *E. coli* strains and additionally confirmed through PCR and sequencing. Subsequently, the microarray was used to type 63 human and animal clinical *E. coli* isolates. All isolates gave signals with control probes and 55/63 (87.3%) were typeable and the results mostly matched clinical diagnosis (where available). Several isolates displayed a novel combination of genes. A more comprehensive system, based on long oligonucleotide probes, was described by Bruant and co-workers [37]. This microarray enabled the detection of 189 *E. coli* virulence genes and 30 antimicrobial resistance genes. After detailed validation with a set of reference strains the microarray was used for a screening of *E. coli* in river waters [38]. 

Microarray for *E. coli* serotyping was developed by Ballmer and co-workers [39]. The probe set targeted *wzx* (O-antigen flippase), *wzy* (O-antigen polymerase) and *fliC* (flagellar monomer) genes and enabled differentiation of epidemiologically most relevant O and H antigens (24 out of >180 and 47 out of 53 respectively). Validation was performed with a set of reference strains and reported sensitivity and specificity were 96% and 90% respectively. Among others, differences were also observed for reported non-motile strains (H-) that showed a clear *fliC* signal on the microarray, indicating that the gene was present but not expressed.

The issue of *Salmonella* spp. typing was addressed by Huehn and co-workers [40]. The probe set targeted genes encoding for pathogenicity, antimicrobial resistance, serotype markers, fimbriae, DNA mobility, metabolic functions and prophages and was validated with set of 23 *Salmonella* isolates and one *E. coli* strain. PCR screening was used as a reference method. Comparison between two sets of data revealed high agreement (96.4%) and the disagreements were mostly due to high homology of mobile elements that could not be distinguished by long oligonucleotide probes. In a follow-up study, Gronlund and co-workers [41] tested the performance of this *Salmonella* spp. typing array in an inter-laboratory evaluation. Four parameters (spotting, DNA extraction, hybridization and wash buffers) were tested, and the hybridization buffer was identified as the most critical factor. This is not surprising since the hybridization buffer (*i.e.*, formamide and salt concentration) has a significant effect on the hybridization stringency. However, it is important to perform such studies in order to ensure robustness and transferability of diagnostic tools.

Different *Salmonella* spp. serotyping microarrays were developed. The Salmonella Premi®Test employs a ligation detection reaction and targets different, undisclosed genomic loci. Wattiau and co-workers reported on two performance studies [42,43]. In the first one 754 *Salmonella* strains belonging to 58 different serovars were analyzed. By classical serotyping 685 tested isolates (90.8%) could be identified. Performance of the Premi®Test was influenced by the quality of DNA extraction (crude extracts *vs.* purified gDNA) and better results (714 typeable isolates, 94.7%) were obtained with purified gDNA. Remaining isolates yielded either non-interpretable (19, 2.5%), dual (16, 2.1%) or wrong (5, 0.6%) results. The second study included 443 *Salmonella* strains. Eighty-four unique profiles were identified with the Premi®Test, whereas classical serotyping identified 62 serovars. These differences were the consequence of the different markers employed by these two typing systems. Another development was described by Tankouo-Sandjong and co-workers [44]. This *Salmonella* spp. serotyping microarray was based on two housekeeping (*gyrB* and *atpD*) and two flagellar (*fliC* and *fljB*) genes and targets two species of *Salmonella* (*S. enterica* and *S. bongori*), the five subspecies of *S. enterica* (II, IIIa, IIIb, IV, VI) and 43 *S. enterica* ssp. *enterica* serovars. This microarray was validated with 57 *Salmonella* strains and 35 blind samples (including mixtures of different serotypes). Even though some cross-hybridization events were observed, high probe redundancy allowed reliable differentiation of targeted serotypes. In addition, a potential for multiple detection was demonstrated, although this was limited by the combination of serotypes present in a mixture. Performance of the microarray was also tested with spiked food samples and sensitivity was shown to depend on the biological pre-enrichment (10^3^ cfu/25 g food after 8h and 1 cfu/25 g food after overnight enrichment).

A more comprehensive *Enterobacteriaceae* typing tool was developed by Friedrich and co-workers [8]. A whole genome comparison was performed with available genomes of *E. coli*, *Shigella* spp., *Salmonella* spp., *Yersinia* spp. and *Klebsiella pneumoniae*. A major problem encountered was the limited genome availability for certain pathotypes (e.g., EAEC or EPEC). The aim was to establish a hierarchical array with 20 probes targeting each designated pathogroup (with detailed differentiation of the “Shigella/*E. coli*” group). However, no targets could be identified for entities “*E. coli*” and “pathogenic *E. coli*” and some pathogroups were targeted by less than 20 probes. In addition to typing probes, antimicrobial resistance gene probes were added. The microarray was thoroughly validated using more than 100 isolates. Subsequently, the microarray was challenged with 92 clinical isolates and the results correlated well with phenotypic characteristics. 

Pathogens are commonly characterized by screening for antimicrobial (antibiotic) resistances. This is particularly the case for clinical pathogens, however antimicrobial typing of environmental (mostly food-borne) pathogens is gaining importance. One of the first reports on the development of an antimicrobial MDM was described by Call and co-workers [45]. PCR amplicons were used as probes targeting 17 *tet* (tetracycline resistance) and *blaTEM-1* (β-lactamase) genes. The microarray was tested with a set of strains belonging to different species and including both *tet+* and *tet-* strains, and results agreed well with the phenotype.

More comprehensive antimicrobial resistance MDMs were later described for gram-positive [46] and gram-negative [47] bacteria. Both systems were developed on the ArrayTube platform and DNA amplification/labeling protocols were selected to enable a high degree of multiplexing (randomly primed polymerization and linear amplification). The MDM developed by Perreten and co-workers [46] targets 90 antimicrobial resistance genes (coverage: 1 to 2 probes per gene). The validation with 36 strains carrying specific antimicrobial resistances allowed testing of the sensitivity and specificity of 125 probes (out of 137) and the microarray results corresponded to the phenotype. Similar results were obtained for multidrug-resistant field isolates. One limitation of this microarray, however, was the inability to distinguish antimicrobial resistances, which arose from single base mutations. Forty-seven antimicrobial resistance genes were targeted with the microarray developed by Batchelor and co-workers [47] and the system was validated with a set of selected reference strains. PCR was used as a reference method and correlation between two methods was 98.8%. Some minor cross-hybridizations were observed (high homology of targeted genes), however, differentiation was not compromised since the resulting patterns were still distinguishable. Interestingly, in most control strains additional genes were detected. Additional tests were performed using 50 *E. coli* (human) and 37 *Salmonella* spp. (human, animal and food) isolates. Phenotype and genotype (microarray) data showed good agreement (70−100%) for approximately 75% of the tested isolates. However, a range of false positive and false negative results were also observed. 

## 4. Conclusions

As demonstrated in this review, recent years witnessed numerous developments of diagnostic microarrays for the detection or typing of pathogens. Even though this review focused on MDMs developed for applications in food, water and environmental analysis (intentionally omitting clinical applications) it does not address all developments, but rather focuses on a few representative ones. Noteworthy, however, is the fact that in spite of the high quantity and mostly good quality of published MDMs their transfer into routine diagnostics is still awaited. Some potential explanations for this hold-up are discussed below.

### 4.1. Specificity and Informational Content

In contrast to clinical applications, where only a group of potential agents has to be considered and potential cross-reactivity with other, related but clinically not-relevant, species is mostly not an issue, MDMs for applications in food, water and environmental analysis have to consider (*i.e.*, differentiate) a much broader spectrum of closely related organisms. This has to be addressed by an appropriate probe design. As already discussed, the 16S rRNA gene is the most commonly used phylogenetic marker with the most comprehensive, available sequence database; however, the high degree of conservation does not allow for unambiguous differentiation on the species level and can consequently give rise to false positive results. Alternative phylogenetic markers (such as *gyrB* or *rpoB*) can alleviate this resolution problem, however, the quality of the probe set is severely influenced by the limited availability of sequences. In order to ensure a good quality and robustness of the system two approaches can be recommended: (i) high probe redundancy; and (ii) *in vitro* validation with both different isolates of targeted species (inclusivity) and a number of closely related, non-targeted organisms (exclusivity). Another issue to be considered is the informational content inherent to the selected marker gene. Universal marker genes (e.g., 16S rDNA, *gyrB* or *rpoB*) provide information about the phylogenetic level of a microbe, but not about its potential pathogenicity (e.g., the analytical result *E. coli* itself is insufficient and further affiliation to EHEC, EPEC or similar is required). This issue can only be addressed through implementation of specific virulence genes. Analogously, typing of the organisms (e.g., patho- or sero-typing) is also dependent on alternative, non-phylogenetic markers. The use of such markers is associated with two major problems: (i) availability of sequence data; and (ii) problems related to multiplex amplification of all targeted markers. 

### 4.2. Sensitivity

Pathogenic organisms in the environment are mostly present in low abundance (low absolute numbers), and are additionally masked by the natural microflora (low relative abundance). Therefore, proposed detection tools have to demonstrate high absolute and relative sensitivity. PCR amplification is one of the most commonly used DNA amplification methods and utilization of gene specific primers allows for specific enrichment of targeted genes thus promoting absolute sensitivity. However, and especially in the case of highly conserved phylogenetic marker genes, conserved primers will ensure amplification of the target gene from all organisms in the sample and might thus negatively influence relative sensitivity (*i.e.*, if the pathogen is present in low abundance in the background of the natural microflora it can be suppressed below detectable levels in course of pre-analytical amplification). Another problem correlated with PCR amplification is the limited multiplexing potential. Therefore, approaches based on conventional PCR amplification are difficult to optimize for systems which target many different genes (e.g., virulence gene-based typing arrays).

As an alternative, whole genome amplification methods can be employed. However, in this case there is no specific enrichment of target regions and thus the sensitivity of the resulting system might be problematic. Lee and co-workers [48] compared the performance of two MDMs for the detection of bacterial pathogens from municipal wastewater. One MDM employed 10 functional genes (targeted by long oligonucleotide probes) and was coupled with whole genome amplification. The second MDM targeted the 23S rRNA gene (short oligonucleotide probes) and was coupled with PCR amplification. The sensitivity of the second (PCR-based) system was found to be 6 orders of magnitude higher (2 × 10^8^*vs.* 1.4 × 10^2^ gene copies respectively). 

An interesting new development was described by Palka-Santini and co-workers [49], who developed a large scale multiplex PCR (LSplex PCR) for co-amplification of 800 different gene regions. Fidelity and sensitivity of this approach were analyzed by comparison with whole genome amplification, and it was found that the sensitivity was improved by a factor of 100 to 1000 with fidelity of >95%. Even though, some of the targets amplified poorly, high redundancy of the target-specific probes on the microarray still allowed for unambiguous detection. A proof-of-principle experiment was performed using clinical material (swabs) and the results obtained by using LSplex PCR followed by microarray hybridization were confirmed by routine microbiological assays. 

Biological pre-enrichment is another sensitivity enhancing method, and the only one that can address the issue of live/dead differentiation, which is of major importance especially when discussing issues of food and water safety. MDMs as well as PCR and whole genome amplification are entirely molecular-based methods and thus detect the presence of targeted DNA regardless of the fact if it comes from a living or dead organism. On the other hand, there is a whole range of disadvantages related to such an approach. Firstly, biological enrichment consumes a lot of time, and duration of the enrichment has a strong influence on the final sensitivity of the system [34,44]. Secondly, biological enrichment has a very limited multiplexing potential, since the combination of nutrients and environmental factors (temperature, oxygen) has to address specific physiological requirements of the target organism [34]. Thirdly, even cultivation-based approaches fail to detect viable but non-culturable bacteria [50]. Another potentially problematic consequence of culture-based sample preparation is the inhibitory effect that that some components of the culture media can have on the subsequent enzymatic reactions [51]. Finally, an important consideration that has to be made when employing a biological pre-enrichment strategy is the proper handling and disposal of the enriched sample containing a high pathogen concentration.

### 4.3. Issues of Live/Dead Differentiation

Another important issue related with the molecular detection of pathogens is the live/dead differentiation which cannot be facilitated through the detection assay itself. As already mentioned, biological pre-enrichment is one approach that could be utilized. However, taking into consideration associated disadvantages (especially limited multiplexing potential) it does not seem to be appropriate for the application with diagnostic microarrays. However, several other promising approaches for integration of the live-dead differentiation into microarray analysis have been suggested. For example, Adamczyk and co-workers [52] reported on the development of the isotope microarrays which allowed linking microbial community structure to its function. Similarly, Bodrossy and co-workers [53] used mRNA-based analysis in order to gain insight into functioning of microbial community. Both approaches seem to be promising, however, RNA-based methods are technically more complex and less robust and therefore their applicability for the routine analysis remains questionable. Pre-treatment with monoazide dyes (in particular propidium monoazide (PMA)) is a commonly used method for the integration of live/dead differentiation in the molecular detection assays (especially PCR-based assays) [54,55]. A recent study by Nocker and co-workers [56] demonstrated that PMA treatment can also be combined with downstream microarray analysis. Furthermore, this study conveys first evidence that the suggested method can be used on environmental samples (spiked water samples). 

### 4.4. Development of the Comprehensive Analytical Chain

Another critical point is the applicability of microarrays for the analysis of the “real-life” samples. Most publications report only on limited proof-of-principle studies and very often lack comprehensive validation with “real-life” samples (for complete overview see Table 1). Major challenges encountered in this phase can be summed up as background effect: (i) low abundance of the pathogen; (ii) effect of the non-targeted background flora; and (iii) effect of the sample matrix. Therefore it is not sufficient to develop a good diagnostic tool per se, but rather a complete analytical chain for the intended application has to be established. This was nicely addressed by Lemarchand and co-workers [57]. In a comprehensive study different protocols for the extraction of nucleic acids from wastewater pellets (initial sample preparation step was low-speed centrifugation) were examined. Tested methods included different (mechanical, chemical and enzymatic) cell lysis protocols in combination with different purification protocols. The quality of each protocol was assessed by analyzing the quantity, purity and degradation level of obtained DNA, as well as the performance in the downstream microarray analysis. Out of ten tested protocols, only three proved to be suitable for the proposed analysis (wastewater samples and microarray analysis). In order to facilitate transfer of the MDMs into routine use, more of such solutions will be necessary. 

It is important to emphasize that the bottlenecks that were identified here are not limited to microarray technology solely, but are related to molecular diagnostic methods in general [58,59].

Since its first publication in 1995 [1], microarray technology underwent significant evolution, both technically, but also in terms of application fields. Nevertheless, it is obvious that further improvements are required in order to facilitate implementation into routine diagnostics. Last few years witnessed a rapid development of new high-throughput sequencing methods and thus the question about the future of microarray technology and its applications arose. In general, each method has its advantages and disadvantages and certain application areas where it is most suitable. Although microarray technology will be replaced by the next-generation sequences in some areas, it is not probable that it will be completely discontinued. Coppee [60] suggested that “these approaches are rather more complementary than mutually exclusive”; and Ledford [61] stated that “improved genetic understanding of disease actually opens up the microarray platform to clinical diagnostics”. 

Taking into consideration the increasing need for ensuring food and water quality and safety (limiting resources, international trade, ageing society) and limitations of currently available methods, it is even more important to rapidly tackle these remaining problems and bottlenecks in order to finally facilitate full transfer of this promising technology from the research laboratories into routine use. 

## Appendix

**Table 1:** Overview of microbial diagnostic microarrays developed for food and water analysis (n.d. = not defined).

Reference		Targeted organisms	Marker gene	Probes	Amplification	Specificity tests	Sensitivity tests	Proof-of-principle	Comments
**Detection MDMs**										
Wang *et al.*, 2007	[ 20]	food-borne	16S rRNA, *invA*, *virA*	short oligonucleotides	PCR	only inclusivity test	10−10^3^ cfu / g food	+/− (bacterial isolates from food)	resolution problem on species level2^nd^ array ( *invA* + *virA*) needed for differentiation of *E. coli/Shigella* spp. and *Salmonella* spp.
Lee *et al.*, 2008	[ 21]	water-borne (38 species, 4 genera, and 1 family)	16S rRNA	short oligonucleotides	PCR	only *in-silico* test	10^3^ cfu (absolute) and 1% (relative)	+ (wastewater samples)	resolution problem on species level
Cremonesi *et al.*, 2009	[ 23]	food-borne (diary products)(15 bacterial groups)	16S rRNA	LDR probes	PCR	inclusivity + limited exclusivity test	6−12 fg gDNA pre-PCR	+ (milk samples)	SNP differentiation through LDR approach
Wang *et al.*, 2009	[ 24]	food-borne (powder infant formula)(10 pathogenic bacteria)	ITS, *wzy*	short oligonucleotides	duplex PCR	inclusivity + exclusivity test	0.001−0.1 ng gDNA pre-PCR1−10 cfu / 25 g PIF (with biological pre-enrichment)	+(powder infant formula samples)	comprehensive *in vitro* validation
Maynard *et al.*, 2005	[ 25]	water-borne	16S rRNA, *cpn60*, *wecE*	short oligonucleotides	PCR (3 rnx)	incomplete inclusivity test	0.1% (corresponding to 10^4^ genomes)	+/− (spiked wastewater gDNA samples)	
Kostic *et al.*, 2010	[ 26]	food- and water-borne	*gyrB*	short oligonucleotides	PCR	inclusivity + exclusivity test	10^4^ cfu (absolute) and 0.1% (relative)1−10 cfu / 25 g food (with biological pre-enrichment)	+(spiked food and water samples)	SNP differentiation through SSELO method
Wilson *et al.*, 2002	[ 30]	18 pathogenic organisms(11 bacteria, 5 RNA viruses, 2 eukaryotes)	142 unique diagnostic regions	short oligonucleotides	multiplex PCR + RT-PCR	inclusivity test	10 fg gDNA pre-PCR2.5% relative	+/− (spiked air gDNA samples)	high density microarray (>50000 probes)
Miller *et al.*, 2009	[ 31]	food- and water-borne(12 pathogenic bacteria)	35 virulence and marker genes	short oligonucleotides	multiplex PCRs (5 rnx, 9- to 10-plex)	inclusivity test	0.1−0.01%	+/− (spiked water gDNA samples)	highly redundant probe set (1−5 genes / pathogen; 8−35 probes / gene)
Kim *et al.*, 2008	[ 32]	food-borne(11 pathogenic bacteria)	pathogen specific DNA-regions (identified by comparative genomics)	long oligonucleotides	WGA	inclusivity + exclusivity test	n.d.	n.d.	only one diagnostic region/pathogen
**Detection/Typing MDMs**										
Call *et al.*, 2001	[ 28]	*E. coli* O157:H7	** *eaeA* , *hylA* , *stx1* , *stx2*	short oligonucleotides	multiplex PCR	inclusivity + limited exclusivity test	10 fg gDNA pre-PCR2.4 × 10^9^ copies of PCR product~100 cfu	+ (spiked chicken carcass rinsates)	
Sergeev *et al.*, 2004	[ 29]	*Listeria* spp., *Campylobacter* spp., *S. aureus, C. perfringens*	** *iap, glyA, fur, ste* genes, *CPT* genes	short oligonucleotides	PCR (8 rnx)	only inclusivity test	n.d.	n.d.	
Peterson *et al.*, 2010	[ 33]	43 pathogenic bacteria	113 virulence genes, 227 antimicrobial resistance genes, metal resistance genes, horizontally transferrable elements	long oligonucleotides	WGA	limited inclusivity test (only 7 targeted organisms)	10^9^ cfu/g (absolute)10^3^ cfu/g (with biological pre-enrichment)	+/− (spiked manure samples)	only one probe/genehighly complex hybridization patterns
Suo *et al.*, 2009	[ 34]	*E. coli* O157:H7, *Salmonella* spp., *L. monocytogenes, C. jejuni*	****14 virulence genes	long oligonucleotides	multiplex PCR (14-plex)	inclusivity test	0.1 pg gDNA pre-PCR	+ (meat samples)	only one probe/gene
Berthet *et al.*, 2008	[ 35]	42 viruses + 50 bacterial species	229 pathogenicity and virulence genes, 390 antimicrobial resistance genes	short oligonucleotides	WGA	incomplete	one genome copy (absolute)>0.01% (relative)	+/− (one wound sample)	high density Affymetrix array (2.56 million probes)
**Typing MDMs**										
Anjum *et al.*, 2007	[ 36]	*E. coli* pathotyping	**virulence and bacteriocin genes	short oligonucleotides	linear	inclusivity test	n.d.	clinical isolates (55/63 typeable)	ArrayTube platform
Bruant *et al.*, 2006	[ 37]	*E. coli* pathotyping	**virulence and antimicrobial resistance genes	long oligonucleotides	WGA	inclusivity test	n.d.	screening of river waters (Hamelin *et al.* , 2007, [ 33 ])	
Ballmer *et al.*, 2007	[ 39]	*E. coli* serotyping	** *wzx, wzy, fliC*	short oligonucleotides	linear	inclusivity test (sensitivity 96%, specificity 90%)	10^6^ genome copies	n.d.	24/118 O-antigens and 47/53 H-antigens
Huehn *et al.*, 2009	[ 40]	*Salmonella* spp. typing	**functional genes	long oligonucleotides	WGA	inclusivity test	n.d.	typing of 4,12:d:- isolates	
Wattiau *et al.*, 2008a,b	[ 42,43]	*Salmonella* spp. serotyping	**undisclosed genomic loci	LDR probes	multiplex PCR	n.d.	n.d.	performance studies (754 and 443 isolates)	commercial productresults not corresponding to Kauffmann-White scheme
Tankouo-Sandjong *et al.*, 2008a	[ 44]	*Salmonella* spp. serotyping	** *atpD, gyrB, fliC, fljB*	short oligonucleotides	PCR (4 rnx)	inclusivity test	10^3^ cfu (absolute)1 cfu/25 g food (with biological pre-enrichment)	panel of blind samples	parallel typing of mixed cultures possible
Friedrich *et al.*, 2010	[ 8]	*Enterobacteriaceae* typing	**pathogroup specific DNA-regions (identified by comparative genomics)	long oligonucleotides	WGA	inclusivity test	n.d.	clinical isolates	complex data analysis algorithms
Call *et al.*, 2003	[ 45]	antimicrobial resistance	*tet, bla_TEM-1_*	PCR amplicons	nick-translation	inclusivity + exclusivity test	n.d.	n.d.	
Peretten *et al.*, 2005	[ 46]	antimicrobial resistance (Gram-positive)	90 antimicrobial resistance genes	short oligonucleotides	linear	inclusivity(125/137 probes tested)	n.d.	n.d.	SNP differentiation not possible
Batchelor *et al.*, 2008	[ 47]	antimicrobial resistance (Gram-negative)	47 antimicrobial resistance genes	short oligonucleotides	linear	inclusivity	n.d.	clinical isolates	discrepancy phenotype *vs.* genotype

## References

[1] Schena M., Shalon D., Davis R.W., Brown P.O. (1995). Quantitative monitoring of gene expression patterns with a complementary DNA microarray. Science.

[2] Bodrossy L., Sessitsch A. (2004). Oligonucleotide microarrays in microbial diagnostics. Curr. Opin. Microbiol..

[3] Call D.R. (2005). Challenges and opportunities for pathogen detection using DNA microarrays. Crit. Rev. Microbiol..

[4] Kostić T., Francois P., Bodrossy L., Schrenzel J., Zourob M., Elwary S., Turner A. (2008). Oligonucleotide and DNA microarrays: Versatile tools for rapid bacterial diagnostics. Principles of Bacterial Detection: Biosensors, Recognition Receptors and Microsystems.

[5] Rasooly A., Herold K.E. (2008). Food microbial pathogen detection and analysis using DNA microarray technologies. Foodborne Pathog. Dis..

[6] Velusamy V., Arshak K., Korostynska O., Oliva K., Adley C. (2010). An overview of foodborne pathogen detection: In perspective of biosensors. Biotechnol. Adv..

[7] Volokhov D.V., Kong H., Herold K., Chizhikov V.E., Rasooly A., Khademhosseini A., Suh K.-Y., Zourob M. (2011). Oligonucleotide microarrays for identification of microbial pathogens and detection of their virulence-associated or drug-resistance determinants. Biological Microarrays: Methods and Protocols.

[8] Friedrich T., Rahmann S., Weigel W., Rabsch W., Fruth A., Ron E., Gunzer F., Dandekar T., Hacker J., Müller T., Dobrindt U. (2010). High-throughput microarray technology in diagnostics of enterobacteria based on genome-wide probe selection and regression analysis. BMC Genomics.

[9] Letowski J., Brousseau R., Masson L. (2004). Designing better probes: Effect of probe size, mismatch position and number on hybridization in DNA oligonucleotide microarrays. J. Microbiol. Methods..

[10] Vora G.J., Meador C.E., Stenger D.A., Andreadis J.D. (2004). Nucleic acid amplification strategies for DNA microarray-based pathogen detection. Appl. Environ. Microbiol..

[11] Baggerly K., Mitra R., Grier R., Medhane D., Lozano G., Kapoor M. (2004). Comparison of sample-labeling techniques in DNA microarray experiments. Anal. Chim. Acta..

[12] (2011). Alere Technologies GmbH Homepage. http://alere-technologies.com/en/products/lab-solutions.html/.

[13] (2011). Legyon Homepage. http://www.legyon.nl/en/our-products/legionellachip.

[14] (2011). Genomica Homepage. http://www.genomica.es/en/in_vitro_diagnostics_products.cfm.

[15] (2011). Luminex Corporation Homepage. http://www.luminexcorp.com/.

[16] Dunbar S.A. (2006). Applications of Luminex® xMAPTM technology for rapid, high-throughput multiplexed nucleic acid detection. Clin. Chim. Acta.

[17] Amann R.I., Ludwig W., Schleifer K.-H. (1995). Phylogenetic identification and *in situ* detection of individual microbial cells without cultivation. Microbiol. Rev..

[18] Santos S.R., Ochman H. (2004). Identification and phylogenetic sorting of bacterial lineages with universally conserved genes and proteins. Environ. Microbiol..

[19] Klappenbach J.A., Saxman P.R., Cole J.R., Schmidt T.M. (2001). rrndb: The ribosomal RNA operon copy number database. Nucleic. Acids. Res..

[20] Wang X.-W., Zhang L., Jin L.-Q., Jin M., Shen Z.-Q., An S., Chao F.-H., Li J.-W. (2007). Development and application of an oligonucleotide microarray for the detection of food-borne bacterial pathogens. Appl. Microbiol. Biotechnol..

[21] Lee D.-Y., Lauder H., Cruwys H., Falletta P., Beaudette L.A. (2008). Development and application of an oligonucleotide microarray and real-time quantitative PCR for detection of wastewater bacterial pathogens. Sci. Total. Environ..

[22] (2011). BLAST. http://blast.ncbi.nlm.nih.gov/Blast.cgi.

[23] Cremonesi P., Pisoni G., Severgnini M., Consolandi C., Moroni P., Raschetti M., Castiglioni B. (2009). Pathogen detection in milk samples by ligation detection reaction-mediated universal array method. J. Dairy Sci..

[24] Wang M., Cao B., Gao Q., Sun Y., Liu P., Feng L., Wang L. (2009). Detection of Enterobacter sakazakii and other pathogens associated with infant formula powder by use of a DNA microarray. J. Clin. Microbiol..

[25] Maynard C., Berthiaume F., Lemarchand K., Harel J., Payment P., Bayardelle P., Masson L., Brousseau R. (2005). Waterborne pathogen detection by use of oligonucleotide-based microarrays. Appl. Environ. Microbiol..

[26] Kostić T., Stessl B., Wagner M., Sessitsch A., Bodrossy L. (2010). Microbial diagnostic microarray for food- and waterborne pathogens. Microb. Biotechnol..

[27] Rudi K., Treimo J., Nissen H., Vegarud G. (2003). Protocols for 16S rDNA array analyses of microbial communities by sequence-specific labelling of DNA probes. Scientific World Journal.

[28] Call D.R., Brockman F.J., Chandler D.P. (2001). Detecting and genotyping Escherichia coli O157:H7 using multiplexed PCR and nucleic acid microarrays. Int. J. Food Microbiol..

[29] Sergeev N., Distler M., Courtney S., Al-Khaldi S.F., Volokhov D., Chizhikov V., Rasooly A. (2004). Multipathogen oligonucleotide microarray for environmental and biodefense applications. Biosens. Bioelectron..

[30] Wilson W.J., Strout C.L., DeSantis T.Z., Stilwell J.L., Carrano A.V., Andersen G.L. (2002). Sequence-specific identification of 18 pathogenic microorganisms using microarray technology. Mol. Cell. Probes..

[31] Miller S.M., Tourlousse D.M., Stedtfeld R.D., Baushke S.W., Herzog A.B., Wick L.M., Rouillard J.M., Gulari E., Tiedje J.M., Hashsham S.A. (2008). *In situ*-synthesized virulence and marker gene biochip for detection of bacterial pathogens in water. Appl. Environ. Microbiol..

[32] Kim H.-J., Park S.-H., Lee T.-H., Nahm B.-H., Kim Y.-R., Kim H.-Y. (2008). Microarray detection of food-borne pathogens using specific probes prepared by comparative genomics. Biosens. Bioelectron..

[33] Peterson G., Bai J., Nagaraja T.G., Narayanan S. (2010). Diagnostic microarray for human and animal bacterial diseases and their virulence and antimicrobial resistance genes. J. Microbiol. Methods..

[34] Suo B., He Y., Paoli G., Gehring A., Tu S.-I., Shi X. (2010). Development of an oligonucleotide-based microarray to detect multiple foodborne pathogens. Mol. Cell Probes..

[35] Berthet N., Dickinson P., Filliol I., Reinhardt A.K., Batejat C., Vallaeys T., Kong K.A., Davies C., Lee W., Zhang S., Turpaz Y., Heym B., Coralie G., Dacheux L., Burguière A.M., Bourhy H., Old I.G., Manuguerra J.-C., Cole S.T., Kennedy G.C. (2008). Massively parallel pathogen identification using high-density microarrays. Microb. Biotechnol..

[36] Anjum M.F., Mafura M., Slickers P., Ballmer K., Kuhnert P., Woodward M.J., Ehricht R. (2007). Pathotyping Escherichia coli by using miniaturized DNA microarrays. Appl. Environ. Microbiol..

[37] Bruant G., Maynard C., Bekal S., Gaucher I., Masson L., Brousseau R., Harel J. (2006). Development and validation of an oligonucleotide microarray for detection of multiple virulence and antimicrobial resistance genes in *Escherichia coli*. Appl. Environ. Microbiol..

[38] Hamelin K., Bruant G., El-Shaarawi A., Hill S., Edge T.A., Fairbrother J., Harel J., Maynard C., Masson L., Brousseau R. (2007). Occurrence of virulence and antimicrobial resistance genes in Escherichia coli isolates from different aquatic ecosystems within the St. Clair river and Detroit river areas. Appl. Environ. Microbiol..

[39] Ballmer K., Korczak B.M., Kuhnert P., Slickers P., Ehricht R., Hachler H. (2007). Fast DNA-serotyping of Escherichia coli by oligonucleotide microarray. J. Clin. Microbiol..

[40] Huehn S., Bunge C., Junker E., Helmuth R., Malorny B. (2009). Poultry-associated Salmonella enterica subsp. enterica serovar 4,12:d:– reveals high clonality and a distinct pathogenicity gene repertoire. Appl. Environ. Microbiol..

[41] Grønlund H., Vigre H., Löfström C., Folling L., Huehn S., Malorny B., Rådström P., Rudi K., Hoorfar J. (2010). Microarray-based genotyping of Salmonella: Inter-laboratory evaluation of reproducibility and standardization potential. Int. J. Food Microbiol..

[42] Wattiau P., Weijers T., Andreoli P., Schliker C., Vander Veken H., Maas H.M.E., Verbruggen A.J., Heck M.E.O.C., Wannet W.J., Imberechts H., Vos P. (2008). Evaluation of the Premi®Test Salmonella, a commercial low-density DNA microarray system intended for routine identification and typing of Salmonella enterica. Int. J. Food Microbiol..

[43] Wattiau P., Van Hessche M., Schlicker C., Vander Veken H., Imberechts H.  (2008). Comparison of classical serotyping and PremiTest assay for routine identification of common Salmonella enterica serovars. J. Clin. Microbiol..

[44] Tankouo-Sandjong B., Sessitsch A., Stralis-Pavese N., Liebana E., Kornschober C., Allerberger F., Hächler H., Bodrossy L. (2008). Development of an oligonucleotide microarray method for Salmonella serotyping. Microb. Biotechnol..

[45] Call D.R., Bakko M.K., Krug M.J., Roberts M.C. (2003). Identifying antimicrobial resistance genes with DNA microarrays. Antimicrob. Agents Chemother..

[46] Perreten V., Vorlet-Fawer L., Slickers P., Ehricht R., Kuhnert P., Frey J.  (2005). Microarray-based detection of 90 antibiotic resistance genes of Gram-positive bacteria. J. Clin. Microbiol..

[47] Batchelor M., Hopkins K.L., Liebana E., Slickers P., Ehricht R., Mafura M., Aarestrup F., Mevius D., Clifton-Hadley F.A., Woodward M.J, Davies R.H., Threlfall E.J., Anjum M.F. (2008). Development of a miniaturised microarray-based assay for the rapid identification of antimicrobial resistance genes in Gram-negative bacteria. Int. J. Antimicrob. Agents..

[48] Lee D.-Y., Shanon K., Beaudette L.A. (2006). Detection of bacterial pathogens in municipal wastewater using an oligonucleotide microarray and real-time quantitative PCR. J. Microbiol. Methods.

[49] Palka-Santini M., Cleven B.E., Eichinger L., Krönke M., Krut O. (2009). Large scale multiplex PCR improves pathogen detection by DNA microarrays. BMC Microbiol..

[50] Oliver J.D. (2010). Recent findings on the viable but nonculturable state in pathogenic bacteria. FEMS Microbiol. Rev..

[51] Rossen L., Norskov P., Holmstrom K., Rasmussen O.F. (1992). Inhibition of PCR by components of food samples, microbial diagnostic assays and DNA-extraction solutions. Int. J. Food Microbiol..

[52] Adamczyk J., Hesselsoe M., Iversen N., Horn M., Lehner A., Nielsen P.H., Schloter M., Roslev P., Wagner M. (2003). The isotope array, a new tool that employs substrate-mediated labeling of rRNA for determination of microbial community structure and function. Appl. Environ. Microbiol..

[53] Bodrossy L., Stralis-Pavese N., Konrad-Köszler M., Weilharter A., Reichenauer T.G., Schöfer D., Sessitsch A. (2006). mRNA-based parallel detection of active methanotroph populations by use of a diagnostic microarray. Appl. Environ. Microbiol..

[54] Nocker A., Cheung C.-Y., Camper A.K. (2006). Comparison of propidium monoazide with ethidium monoazide for differentiation of live *vs*. dead bacteria by selective removal of DNA from dead cells. J. Microbiol. Methods..

[55] Varma M., Field R., Stinson M., Rukovets B., Wymer L., Haugland R. (2009). Quantitative real-time PCR analysis of total and propidium monoazide-resistant fecal indicator bacteria in wastewater. Water Res..

[56] Nocker A., Mazza A., Masson L., Camper A.K., Brousseau R. (2009). Selective detection of live bacteria combining propidium monoazide sample treatment with microarray technology. J. Microbiol. Methods..

[57] Lemarchand K., Berthiaumea F., Maynard C., Harel J., Payment P., Bayardelle P., Masson L., Brousseau R. (2005). Optimization of microbial DNA extraction and purification from raw wastewater samples for downstream pathogen detection by microarrays. J. Microbiol. Methods..

[58] Lauri A., Mariani P.O. (2009). Potentials and limitations of molecular diagnostic methods in food safety. Genes Nutr..

[59] Girones R., Ferrus M.A., Alonso J.L., Rodriguez-Manzano J, Calgua B., Correa Ade A., Hundesa A., Carratala A., Bofill-Mas S. (2010). Molecular detection of pathogens in water—The pros and cons of molecular techniques. Water Res..

[60] Coppee J.-Y. (2008). Do DNA microarrays have their future behind them?. Microbes Infect..

[61] Ledford H. (2008). The death of microarrays?. Nature.

